# Salt sensitivity and myocardial fibrosis: unraveling the silent cardiovascular remodeling

**DOI:** 10.3389/fphar.2025.1626492

**Published:** 2025-06-13

**Authors:** Katongo Hope Mutengo, Owen Ngalamika, Annet Kirabo, Sepiso K. Masenga

**Affiliations:** ^1^ Department of Internal Medicine, Ministry of Health, Monze Mission Hospital, Monze, Zambia; ^2^ Department of Internal Medicine, School of Medicine, University of Zambia, Lusaka, Zambia; ^3^ Department of Cardiovascular Science and Metabolic diseases, Livingstone Center for Prevention and Translational Science, Livingstone, Zambia; ^4^ Dermatology and Venereology Section, School of Medicine, University of Zambia, Lusaka, Zambia; ^5^ Department of Medicine, Division of Clinical Pharmacology, Vanderbilt University Medical Center, Nashville, TN, United States; ^6^ Vanderbilt Center for Immunobiology, Vanderbilt Institute for Infection, Immunology and Inflammation, Vanderbilt Institute for Global Health, Vanderbilt University Medical Center, Nashville, TN, United States; ^7^ Department of Molecular Physiology and Biophysics, Vanderbilt University Medical Center, Nashville, TN, United States; ^8^ Department of Pathology, Mulungushi University, Livingstone, Zambia

**Keywords:** salt, sensitivity, myocardial, fibrosis, remodeling, HFPEF, ^23^Na-MRI

## Abstract

Salt sensitivity is a well-recognized contributor to cardiovascular risk, traditionally linked to elevated blood pressure. However, emerging evidence suggests that high dietary sodium may also promote myocardial fibrosis through non-hemodynamic mechanisms, including the activation of redox-sensitive and profibrotic pathways. Despite growing mechanistic insights, the connection between salt sensitivity and myocardial fibrosis remains underexplored, particularly in human studies. This review synthesizes current experimental and translational evidence linking dietary salt intake to myocardial fibrosis, with a focus on molecular signaling cascades, tissue sodium compartmentalization, and the clinical implications of salt-sensitive physiology. We discuss the relevance of these mechanisms to the development of diastolic dysfunction and their potential contribution to heart failure with preserved ejection fraction (HFpEF). In addition, we highlight findings from animal models and the emerging application of sodium magnetic resonance imaging (^23^Na-MRI) as a novel imaging tool for visualizing myocardial sodium overload and its association with fibrotic remodeling. Finally, we explore future therapeutic strategies that extend beyond traditional antihypertensives, including mineralocorticoid receptor antagonists (MRAs), angiotensin receptor blockers (ARBs), sodium-glucose cotransport 2 (SGLT2) inhibitors, and sodium-modulating interventions. Together, these insights offer new opportunities for early detection and targeted treatment in salt-sensitive cardiovascular disease.

## 1 Introduction

Salt sensitivity is a dynamic physiological trait observed across species, including humans, whereby an individual’s blood pressure fluctuates in direct response to changes in dietary sodium (salt) intake (Weinberger, 1996; Majid et al., 2015; Elijovich et al., 2016; Afolabi et al., 2024). It is estimated that approximately 30%–50% of hypertensive individuals and a significant proportion of normotensive individuals exhibit some degree of salt sensitivity (Morris et al., 1999; Majid et al., 2015; Afolabi et al., 2024). While the role of sodium in blood pressure regulation is well-established, emerging evidence suggests that salt sensitivity may contribute to myocardial fibrosis through mechanisms beyond blood pressure elevation (Kagiyama et al., 2007; Ferreira et al., 2010; Huang et al., 2024). Myocardial fibrosis, marked by excess extracellular matrix (ECM) deposition, contributes to cardiac remodeling and increases the risk of heart failure with preserved ejection fraction (HFpEF) (Ding et al., 2020; Díez et al., 2020; Schiau et al., 2021).

Animal studies have provided intriguing evidence linking salt sensitivity to myocardial fibrosis (Kagiyama et al., 2007; Ferreira et al., 2010; Huang et al., 2024), demonstrating that chronic high-sodium intake exacerbates collagen deposition and interstitial fibrosis in the heart. Several mechanisms have been proposed to explain this relationship, including inappropriate activation of the renin-angiotensin-aldosterone system (RAAS), oxidative stress, inflammation, endothelial dysfunction, and altered sodium handling at the cellular level (Fielitz et al., 2001; Sopel et al., 2011; Mascolo et al., 2021). Notably, in salt-sensitive individuals, aldosterone levels often remain inappropriately high despite sodium loading, a phenomenon that promotes fibroblast activation and collagen synthesis via mineralocorticoid receptor - mediated pathways (Sopel et al., 2011; Fujita, 2014).

Beyond RAAS activation, excessive sodium intake triggers proinflammatory and profibrotic signaling cascades in the myocardium (Kagiyama et al., 2007). These include endothelial glycocalyx degradation (Sembajwe et al., 2023), and upregulation of transforming growth factor-beta (TGF-β1) - a master regulator of fibrosis (Fielitz et al., 2001; Biernacka et al., 2011; Bakalenko et al., 2024). Additionally, chronic sodium exposure drives macrophage polarization toward an alternatively activated M2 phenotype. While M2 macrophages are primarily associated with anti-inflammation and tissue repair, persistent activation in a high-sodium environment shifts them toward a pro-inflammatory and pro-fibrotic M1 phenotype (Fehrenbach and Mattson, 2020), ultimately contributing to myocardial fibrosis. Additionally, High sodium exposure may lead to intracellular sodium accumulation, mitochondrial dysfunction, and activation of the NOD-like receptor family pyrin domain-containing 3 (NLRP3) inflammasome, all of which contribute to myocardial stiffening and pathological remodeling (Pitzer et al., 2022).

Despite these insights, most of the current understanding of sodium-induced myocardial fibrosis comes from animal models (Kagiyama et al., 2007, 2007; Wendt et al., 2009; Ferreira et al., 2010; Huang et al., 2024). Human studies remain scarce, leaving a critical gap in our ability to translate these findings into clinical practice. While animal models demonstrate that sodium loading exacerbates myocardial fibrosis (Wendt et al., 2009; Ferreira et al., 2010), whether these mechanisms are similar in humans, particularly in salt-sensitive individuals, remains largely unknown. Moreover, traditional clinical studies often focus solely on the blood pressure effects of sodium, overlooking its direct impact on myocardial structure and fibrosis. This review aims to synthesize current evidence on the relationship between salt sensitivity and myocardial fibrosis, explore the underlying molecular mechanisms, and discuss novel diagnostic and therapeutic strategies. Given its rising prevalence and link to cardiovascular risk, understanding salt sensitivity’s role in myocardial fibrosis may inform targeted therapies and early prevention.

**Novelty and rationale for studying salt sensitivity and myocardial fibrosis**

Despite growing recognition of the link between salt sensitivity and myocardial fibrosis, several critical gaps remain:

1. The independent role of sodium beyond blood pressure remains unclear - Most clinical studies focus on the hemodynamic effects of sodium, but could sodium-mediated fibrosis occur even before hypertension develops?
2. Early detection remains elusive - Conventional diagnostic tools often fail to detect early fibrosis before symptomatic heart failure develops. Could novel imaging modalities such as sodium-23 magnetic resonance imaging (^23^Na-MRI) on cardiac MRI help identify preclinical fibrosis in salt-sensitive individuals?
3. Salt-sensitive individuals may represent an under-recognized phenotype of HFpEF - Given the high prevalence of HFpEF in salt-sensitive patients, could myocardial fibrosis serve as an early predictor of HFpEF progression in this population?
4. Therapeutic strategies remain undefined - While mineralocorticoid receptor antagonists (MRAs), angiotensin receptor blockers (ARBs), sodium-glucose cotransporter-2 (SGLT2) inhibitors, and dietary sodium restriction are potential interventions, their effectiveness in reversing salt-induced fibrosis remains largely unexplored.

### 1.1 Pathophysiological mechanisms linking salt sensitivity and myocardial fibrosis

#### 1.1.1 Sodium and myocardial remodeling

Sodium homeostasis is a tightly regulated physiological process, with intracellular sodium concentrations maintained at low levels (approximately 10–15 mmol/L) relative to the extracellular compartment (∼140 mmol/L) (ScienceDirect Topics, 2006). In healthy myocardium, the intracellular sodium gradient is essential for normal excitation-contraction coupling, myocardial metabolism, and cellular viability (ScienceDirect Topics, 2006).

In the setting of salt sensitivity, however, there is a dysregulation of sodium handling at the tissue level. Salt-sensitive individuals exhibit increased tissue sodium accumulation - not merely in plasma or interstitial spaces, but within glycosaminoglycan-bound reservoirs in the skin, vasculature, and myocardium, independent of volume status or extracellular edema (Kopp et al., 2013; Schneider et al., 2017). This sodium retention occurs without commensurate water retention, rendering it invisible to conventional imaging or hemodynamic measurements. In the myocardium, elevated intracellular sodium levels may lead to mitochondrial dysfunction, increased oxidative stress, and activation of fibrotic signaling cascades (Christa et al., 2024), thus laying the groundwork for diffuse myocardial fibrosis in the absence of overt hypertrophy or volume overload.

While traditionally viewed through the lens of volume expansion and blood pressure elevation, dietary sodium exerts direct effects on the myocardium that extend beyond its hemodynamic consequences (Li et al., 2021). In salt-sensitive mammals, excessive sodium intake contributes not only to hypertension but also to subclinical cardiac remodeling (Hayakawa et al., 2015). This remodeling is increasingly recognized as a fibroinflammatory process, driven by a complex interplay between sodium accumulation, altered cellular signaling, and immune activation (Hayakawa et al., 2015).

Experimental models have shown that a high-sodium diet markedly accelerates cardiac injury through the upregulation of components of the renin–angiotensin system (RAS) (Hayakawa et al., 2015), even in the absence of systemic RAAS activation. In salt-sensitive states, dietary sodium loading has been observed to stimulate cardiac expression of the (pro)renin receptor (PRR) and the angiotensin II type 1 receptor (AT1R) (Hayakawa et al., 2015). This occurs via local increases in prorenin, renin, and angiotensinogen - key precursors in the intratissue RAS cascade (Nguyen et al., 2002). The binding of prorenin or renin to PRR enhances local angiotensin II production and directly initiates intracellular signaling independent of enzymatic activity, contributing to maladaptive remodeling of the heart (Hayakawa et al., 2015).

These molecular changes are accompanied by the activation of several downstream intracellular pathways, notably the extracellular signal-regulated kinases (ERK1/2) (Zhang et al., 2024b), transforming growth factor-beta 1 (TGF-β1) (Fielitz et al., 2001), and p38 mitogen-activated protein kinase (p38MAPK) (Zhang et al., 2003; Park et al., 2007). Each of these pathways plays a key role in the fibrotic transformation of cardiac tissue. The activation of these pathways via the PRR and AT1R potentially contribute to myocardial fibrosis through several mechanisms such as myofibroblast differentiation and upregulation of profibrotic genes.

##### 1.1.1.1 ERK1/2 activation

**FIGURE 1:**
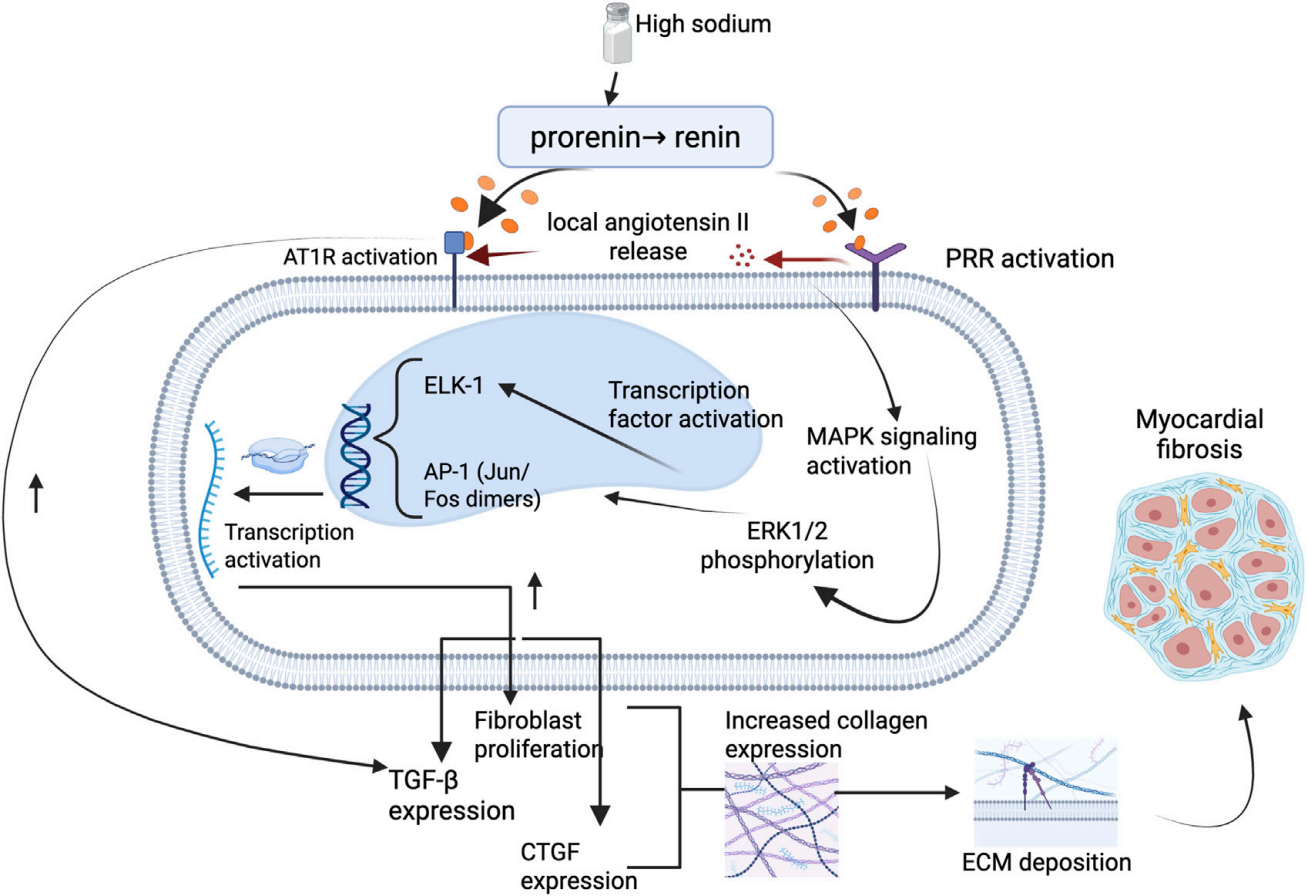
Illustration on the molecular pathway of salt sensitivity leading to myocardial fibrosis. High dietary sodium promotes activation of the PRR, leading to intracellular MAPK signaling via ERK1/2 phosphorylation. This cascade activates key transcription factors including ELK-1 and AP-1 (Jun/Fos dimers), resulting in upregulation of TGF-β and CTGF, increased fibroblast proliferation, and collagen gene expression. The resultant ECM deposition contributes to myocardial fibrosis. This pathway may occur independently of AT1R signaling and underscores a non-hemodynamic mechanism linking sodium excess to structural cardiac remodeling. Abbreviations: PRR, (pro)renin receptor; AT1R, angiotensin II type 1 receptor; ERK1/2, extracellular signal-regulated kinase 1/2; MAPK, mitogen-activated protein kinase; ELK-1, Ets-like kinase 1; AP-1, activator protein-1; TGF-β, transforming growth factor-beta; CTGF, connective tissue growth factor; ECM, extracellular matrix. Figures were created by authors in biorender.com.

Binding of prorenin or renin to PRR initiates a cascade of intracellular signaling events that are independent of the enzymatic conversion of angiotensinogen to angiotensin I (Hayakawa et al., 2015). This ligand–receptor interaction induces conformational changes in PRR that lead to the recruitment and activation of the MAPK pathway, particularly the ERK1/2 kinases (Zhang et al., 2024b). Activation of ERK1/2 via phosphorylation has been identified as a critical step in promoting pathological cardiac remodeling through its downstream effects on profibrotic gene expression and fibroblast function (Hayakawa et al., 2015; Gilbert et al., 2021) (Figure 1).

Once phosphorylated, ERK1/2 translocates to the nucleus, where it activates several transcription factors, most notably Ets-like kinase 1 (ELK-1) (Zhang et al., 2024b) and activator protein-1 (AP-1) (Vidyasagar et al., 2012, p. 1). ELK-1 is a nuclear transcription factor belonging to the E26 transformation-specific (ETS) domain family, which is known to regulate genes involved in cell proliferation, differentiation, and survival (Janknecht et al., 1994). In the context of myocardial remodeling, ERK1/2-mediated phosphorylation of ELK-1 enhances the transcription of genes that promote fibroblast proliferation and collagen synthesis (Gilbert et al., 2021). Similarly, AP-1, formed by Jun and Fos proteins, is activated via ERK1/2 signaling and governs the expression of genes linked to inflammation, ECM remodeling, and fibrosis (Ye et al., 2014; Averill-Bates, 2024). Through these transcriptional programs, ERK1/2 signaling leads to the upregulation of profibrotic mediators such as TGF-β1 and connective tissue growth factor (CTGF), both of which further stimulate fibroblast activation and ECM deposition (Madala et al., 2012; Averill-Bates, 2024).

Experimental studies support this mechanism. Nguyen et al. first described that PRR activation can lead to ERK1/2 phosphorylation independently of angiotensin II, establishing PRR as more than a passive binding site for prorenin (Nguyen et al., 2002). Subsequent work has shown that high sodium and aldosterone exposure further enhance PRR expression and potentiate ERK1/2 signaling, promoting fibrotic gene expression in cardiac tissues (Gilbert et al., 2021; Zhang et al., 2024b). In mouse models, activation of this pathway is associated with increased myocardial collagen content and stiffness, features characteristic of pathological fibrosis (Chatzifrangkeskou et al., 2016). Notably, this mechanism appears to operate independently of classical AT1R signaling, as evidenced by persistent fibrosis in angiotensin II type 1a receptor knockout models exposed to aldosterone and high sodium, suggesting alternative, ERK1/2-centric routes of cardiac injury (Nguyen et al., 2002).

Taken together, these findings illustrate how PRR-mediated ERK1/2 activation serves as a critical bridge between neurohormonal stimulation and structural cardiac remodeling as shown in Figure 1. The involvement of transcription factors such as ELK-1 and AP-1 underscores the transcriptional reprogramming that underlies fibroblast proliferation and matrix accumulation in the setting of salt-sensitivity. Understanding this pathway not only highlights the complexity of salt-induced myocardial fibrosis but also points to potential therapeutic targets beyond the conventional renin-angiotensin system.

##### 1.1.1.2 TGF-β upregulation

Activation of PRR and AT1R enhances TGF-β expression, a central mediator of fibrosis that induces fibroblast-to-myofibroblast differentiation and stimulates collagen synthesis, leading to increased ECM deposition (Border and Noble, 1998). TGF-β is a multifunctional cytokine and a master regulator of fibrosis. It orchestrates the transition of quiescent fibroblasts into activated myofibroblasts, which are the primary effector cells responsible for collagen synthesis and ECM expansion in fibrotic tissue (Biernacka et al., 2011). Concurrently, AT1R activation by angiotensin II enhances the expression of TGF-β both directly and indirectly via reactive oxygen species (ROS) production, nicotinamide adenine dinucleotide phosphate (NADPH) oxidase activation, and stimulation of proinflammatory cytokines (Pendergrass et al., 2009). This creates a feedback loop where TGF-β further promotes AT1R expression and receptor sensitivity, amplifying the fibrotic response.

**FIGURE 2:**
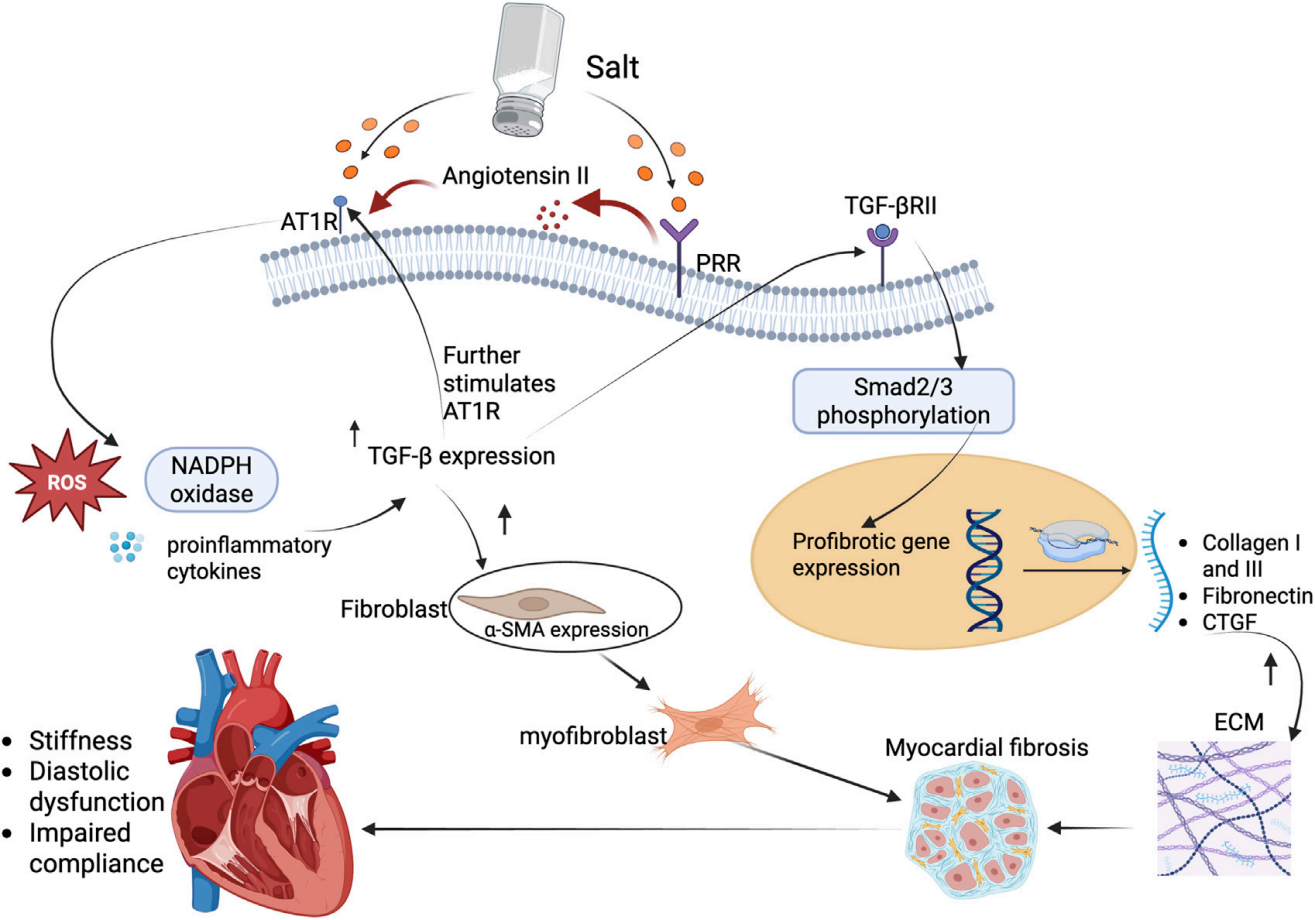
TGF-β profibrotic signaling leading to myocardial fibrosis in salt-sensitive states. High dietary salt activates both PRR and AT1R pathways, leading to increased production and release of TGF-β, a key profibrotic cytokine. AT1R stimulation enhances TGF-β expression through redox-sensitive mechanisms, including ROS generation and inflammatory cytokine signaling. PRR activation further amplifies angiotensin II levels, reinforcing TGF-β upregulation. Once secreted, TGF-β binds to its receptor, TGF-βRII, triggering phosphorylation of Smad2/3 and nuclear translocation of the Smad complex. This drives transcription of profibrotic genes such as collagen I/III, fibronectin, and CTGF. Concurrently, TGF-β promotes fibroblast differentiation into α-SMA-expressing myofibroblasts, resulting in ECM accumulation and progressive myocardial fibrosis. The resulting structural changes impair ventricular compliance, contributing to diastolic dysfunction and clinical HFpEF phenotypes in salt-sensitive individuals. Abbreviations: AT1R, Angiotensin II type 1 receptor; PRR, (pro)renin receptor; TGF-β, transforming growth factor-beta; TGF-βRII, transforming growth factor-beta receptor II; Smad2/3, small mothers against decapentaplegic homolog 2/3; ROS, reactive oxygen species; NADPH, nicotinamide adenine dinucleotide phosphate; α-SMA, alpha-smooth muscle actin; CTGF, connective tissue growth factor; ECM, extracellular matrix. Figures were created by authors in biorender.com.

TGF-β exerts its canonical effects primarily through the Smad2/3 signaling axis (Biernacka et al., 2011). Once secreted, TGF-β binds to its receptor (TGF-βRII), leading to phosphorylation of receptor-regulated Smads (Tzavlaki and Moustakas, 2020). These Smads form complexes with Smad4 and translocate into the nucleus, where they drive the transcription of key profibrotic genes such as collagen type I and III, fibronectin, and CTGF (Yang et al., 2009; Biernacka et al., 2011). These ECM components accumulate in the interstitial and perivascular spaces, resulting in increased myocardial stiffness, impaired compliance, and diastolic dysfunction (Ding et al., 2020) (Figure 2).

Notably, TGF-β also induces the expression of α-smooth muscle actin (α-SMA) in cardiac fibroblasts, promoting their differentiation into myofibroblasts (Hu et al., 2003; Bakalenko et al., 2024). These cells exhibit contractile properties and resistance to apoptosis, which makes their presence persistent in fibrotic tissue. Moreover, PRR and AT1R activation have been shown to potentiate non-canonical TGF-β signaling via p38MAPK, JNK, and RhoA/ROCK pathways (Murphy et al., 2015; Gao et al., 2016; Qi et al., 2020; Lopez-Lopez et al., 2021; Zhang et al., 2024b), further amplifying the fibrotic milieu even in the presence of TGF-β-independent stimuli.

Research has shown that chronic administration of angiotensin II induces TGF-β1 protein expression in the myocardium (Campbell and Katwa, 1997), which plays a pivotal role in myocardial fibrosis by promoting fibroblast proliferation and ECM deposition. On the other hand, mice lacking angiotensin II type 1a receptor or treated with AT1R blockers such as losartan show reduced cardiac fibrosis, not solely due to blood pressure lowering, but due to attenuated TGF-β-mediated profibrotic signaling (Spurney et al., 2011). However, in some models, aldosterone and sodium together still induce fibrosis even when angiotensin II type 1a receptor is genetically deleted (Kagiyama et al., 2007). This suggests that PRR-TGF-β signaling can operate independently of AT1R under certain conditions, possibly via ERK1/2 pathways.

Overall, the coordinated upregulation of AT1R and TGF-β in response to PRR and angiotensin II stimulation creates a robust profibrotic network within the myocardium. This not only drives collagenous ECM accumulation, but also alters cellular phenotype of cardiac fibroblasts, contributing to the structural and functional deterioration characteristic of salt-sensitive hypertensive heart disease.

##### 1.1.1.3 p38MAPK activation

**FIGURE 3:**
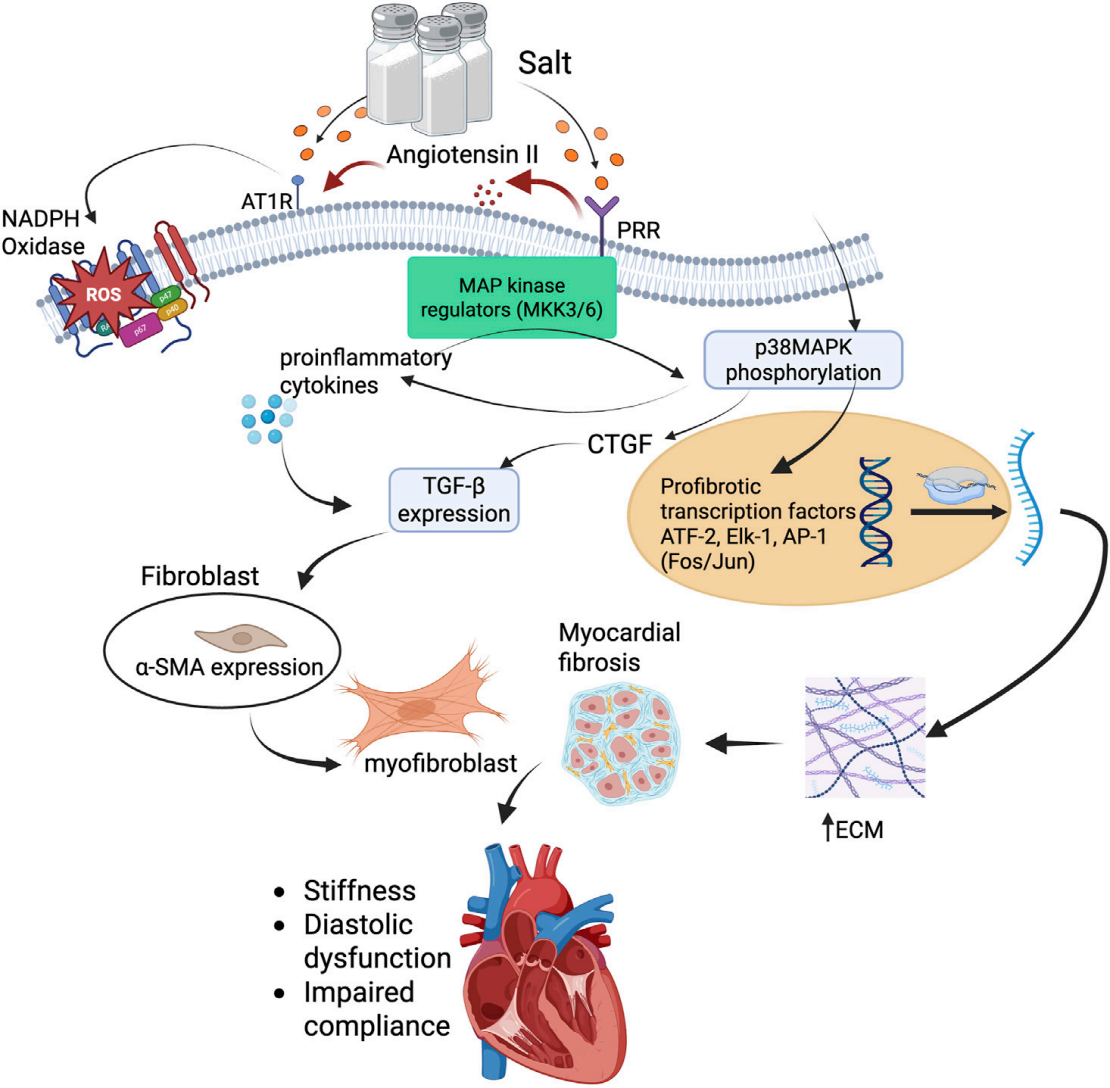
p38MAPK-mediated profibrotic signaling cascade in salt-sensitive myocardial fibrosis. High salt intake stimulates PRR and AT1R activation through increased renin-angiotensin system activity. PRR engagement activates MAP kinase regulators (MKK3/6), leading to phosphorylation of p38MAPK. This triggers downstream activation of profibrotic transcription factors, including ATF-2, ELK-1, and AP-1 (Fos/Jun), which promote transcription of TGF-β and CTGF. Concurrently, AT1R activation promotes ROS generation via NADPH oxidase, driving inflammation and enhancing TGF-β expression. TGF-β induces fibroblast-to-myofibroblast transition via α-SMA expression and promotes ECM production, including collagen and fibronectin. The accumulation of ECM leads to myocardial fibrosis, resulting in increased ventricular stiffness, diastolic dysfunction, and impaired compliance. This pathway illustrates a mechanistic link between high salt intake, MAPK signaling, and structural cardiac remodeling. Abbreviations: PRR, (pro)renin receptor; AT1R, angiotensin II type 1 receptor; ROS, reactive oxygen species; NADPH, nicotinamide adenine dinucleotide phosphate; TGF-β, transforming growth factor-beta; CTGF, connective tissue growth factor; MAPK, mitogen-activated protein kinase; MKK3/6, MAP kinase kinase 3/6; p38MAPK, p38 mitogen-activated protein kinase; ATF-2, activating transcription factor 2; ELK-1, Ets-like kinase 1; AP-1, activator protein-1; α-SMA, alpha-smooth muscle actin; ECM, extracellular matrix. Figures were created by authors in biorender.com.

Activation of the p38MAPK pathway is a well-characterized cellular response to stress stimuli, including oxidative stress, mechanical stretch, and proinflammatory cytokines (Kyriakis and Avruch, 2012). These are all prevalent in the setting of salt-sensitive hypertension and RAAS overactivation. Both PRR and AT1R activation have been shown to stimulate p38MAPK signaling (Stockand and Meszaros, 2003; Lee et al., 2004; Gao et al., 2016), making this pathway a critical node in the convergence of hemodynamic, hormonal, and inflammatory insults that promote myocardial fibrosis (Figure 3).

Upon activation by prorenin or Angiotensin II, PRR and AT1R engage intracellular signaling cascades involving redox-sensitive pathways. These include NADPH oxidase-derived ROS generation, which modulates kinase activity, particularly through the activation of upstream MAP kinase regulators like MKK3/6, culminating in the phosphorylation of p38MAPK and propagation of fibrotic and inflammatory responses (Nguyen et al., 2002; Gui et al., 2012; Nguyen Dinh Cat et al., 2013). This leads to amplified downstream pathways that mediate vascular inflammation, hypertrophy, and fibrosis. Once activated, p38MAPK translocate into the nucleus and phosphorylates a range of transcription factors, including ATF-2, Elk-1, and components of the AP-1 complex (e.g., *Fos, Jun*) (Janknecht et al., 1994; Natarajan et al., 1999; Zhang and LIU, 2002; Kyriakis and Avruch, 2012; Zhang et al., 2024b). These transcription factors then upregulate genes involved in both inflammation and fibrosis. Notably, p38MAPK activation leads to enhanced expression of proinflammatory cytokines such as tumor necrosis factor-alpha (TNF-α), interleukin-1 beta (IL-1β), and interleukin-6 (IL-6) (Ganguly et al., 2023; Zhang et al., 2024b), which act locally to further recruit immune cells and perpetuate tissue injury.

From a fibrotic standpoint, p38MAPK signaling also promotes the expression of CTGF (Lee et al., 2004), a key downstream mediator of TGF-β1, resulting in increased ECM deposition. Interestingly, p38MAPK not only works in tandem with TGF-β signaling but can also function independently to drive fibrotic gene expression (Stockand and Meszaros, 2003; Lee et al., 2004; Kyriakis and Avruch, 2012; Zhang et al., 2024b), especially under conditions of mechanical stress or oxidative injury. Additionally, p38MAPK activation supports the differentiation of cardiac fibroblasts into α-SMA-positive myofibroblasts (Gui et al., 2012), which are central to the development and maintenance of fibrotic lesions (Figure 3).

Experimental studies have supported these mechanisms. In a mouse model of myocardial infarction, selective inhibition of p38MAPK significantly improved cardiac function and reduced fibrosis, with effects comparable to ACE inhibition - highlighting p38MAPK as a key driver of post-infarction remodeling (Liu et al., 2005). Likewise, SB203580, a potent p38MAPK inhibitor, reduces the profibrotic fibroblast-to-myofibroblast transition associated with asthma, indicating its potential in mitigating fibrosis (Paw et al., 2021). Further evidence from salt-loading models demonstrates that dietary sodium can activate both p38 and ERK1/2 MAPK cascades via a tetraethylammonium (TEA)-sensitive pathway (Ying and Sanders, 2002), promoting glomerular TGF-β1 and NOS3 expression - effects that were significantly attenuated by MAPK inhibitors SB203580 and PD-098059. This effect reinforces the salt-sensitive physiology associated with this profibrotic pathway.

Importantly, activation of p38MAPK also induces mitochondrial dysfunction and increases ROS production (Lopez-Lopez et al., 2021), which further amplifies redox-sensitive profibrotic signaling loops. In this context, p38MAPK serves not only as a mediator of immediate stress responses but also as a chronic driver of structural myocardial remodeling. The crosstalk between PRR, AT1R, and p38MAPK therefore represents a potent signaling triad in the pathogenesis of myocardial fibrosis, with implications for early intervention and targeted therapy.

Collectively, these molecular events initiated by a high-sodium diet and mediated through PRR and AT1R activation culminate in myocardial fibrosis, characterized by excessive deposition of ECM components, disruption of normal cardiac architecture, and impairment of cardiac function. Understanding these pathways provides insights into how dietary sodium influences cardiac remodeling resulting in conditions such as HFpEF and highlights potential therapeutic targets for preventing salt-induced cardiac damage.

#### 1.1.2 Clinical heart failure with preserved ejection fraction phenotypes and their overlap with salt sensitivity

HFpEF is now widely acknowledged as a heterogeneous syndrome rather than a uniform disease entity. Recent phenomapping studies (Shah et al., 2015; Lewis et al., 2017; Bonfioli et al., 2025) have identified several distinct clinical subtypes of HFpEF. One of the most compelling observations from recent HFpEF research is the recognition of distinct clinical phenotypes, several of which exhibit pathophysiological features that significantly overlap with salt sensitivity (Ferreira et al., 2010; Elijovich et al., 2016; Kitzman and Shah, 2016; AlGhatrif et al., 2017; Kirkman et al., 2021; Sotomi et al., 2021; Afolabi et al., 2024; Bailey and Dhaun, 2024; Zhang et al., 2024a). Among these include, the obese-inflammatory HFpEF phenotype (Kitzman and Shah, 2016), hypertensive aging HFpEF phenotype (AlGhatrif et al., 2017; Zhang et al., 2024a), renal dysfunction-driven phenotype (Kirkman et al., 2021), and the female predominant HFpEF phenotype (Sotomi et al., 2021). These phenotypes offer a clinically meaningful framework for understanding the heterogeneity of HFpEF and provide a basis for mechanistic exploration. In the following section, we examine each phenotype in detail, highlighting shared and distinct pathways, particularly those intersecting with salt sensitivity and myocardial fibrosis.

##### 1.1.2.1 Obese-inflammatory HFpEF phenotype

This subtype, is characterized by chronic low-grade inflammation, endothelial dysfunction, and systemic oxidative stress (Kitzman and Shah, 2016; Verma et al., 2024). These same pathways are central to the salt-sensitive state, where excessive sodium intake or retention enhances adipose tissue inflammation and further activates mineralocorticoid receptors (Kagiyama et al., 2007; Lopez-Lopez et al., 2021; Pitzer et al., 2022), even in normotensive individuals, creating a convergence of metabolic and ionic stressors that accelerate fibrotic myocardial remodeling. Animal studies have demonstrated that salt loading in obese models accelerates myocardial fibrosis and impairs diastolic function, underscoring the biological plausibility of this overlap (Aoyama et al., 2020).

**FIGURE 4:**
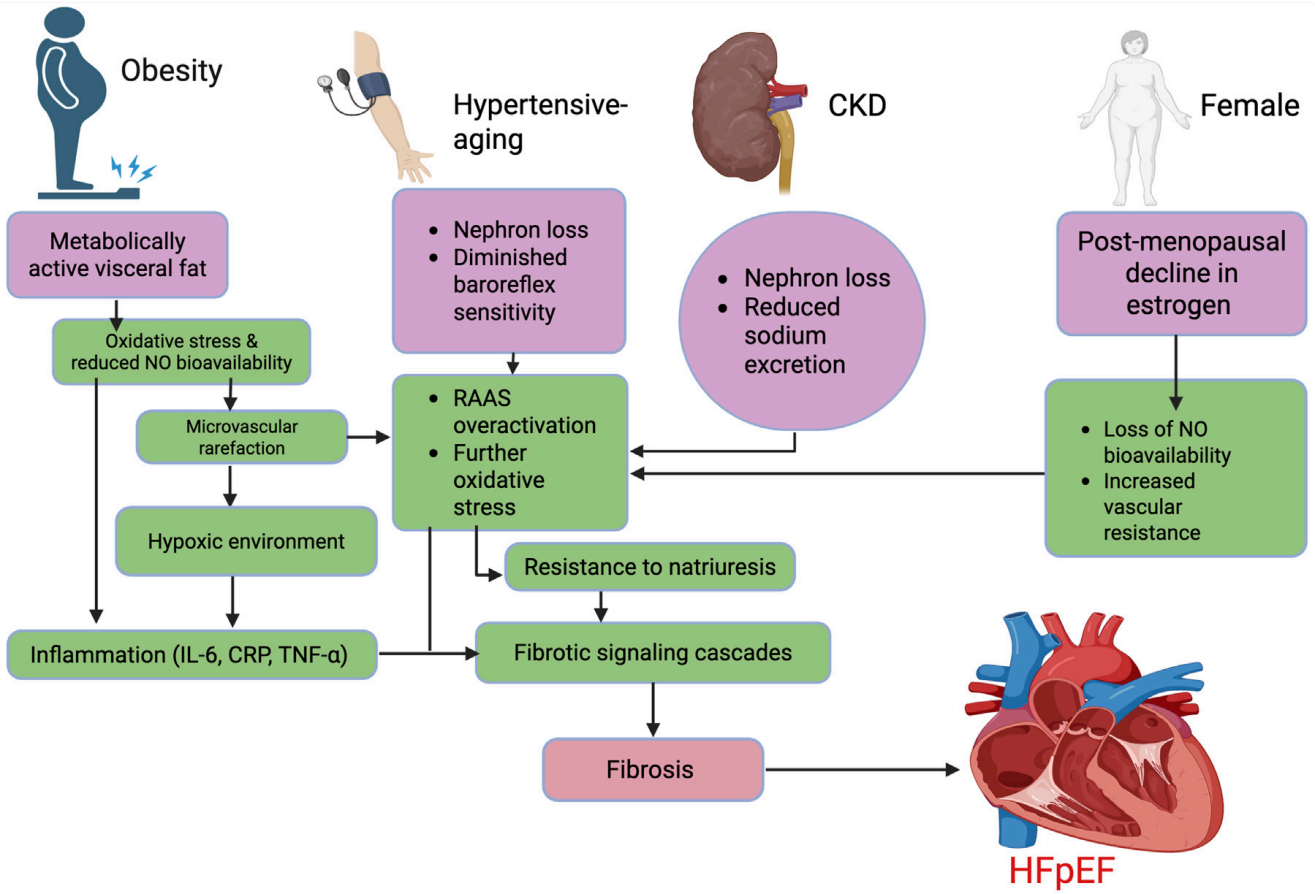
Mechanistic pathways underlying major HFpEF phenotypes. The flow chart outlines four key phenotypes contributing to HFpEF: obesity, hypertensive-aging, CKD, and female sex. Each triggers overlapping mechanisms, including reduced NO bioavailability, inflammation, microvascular dysfunction, oxidative stress, and RAAS activation, leading to fibrotic signaling and myocardial stiffness. These distinct yet converging pathways ultimately drive fibrosis and diastolic dysfunction characteristic of HFpEF. Abbreviations: HFpEF, heart failure with preserved ejection fraction; IL-6, interleukin-6; CRP, C-reactive protein; TNF-α, tumor necrosis factor-alpha; NO, nitric oxide; RAAS, renin–angiotensin–aldosterone system; CKD, chronic kidney disease. Figures were created by authors in biorender.com.

Adipose tissue, particularly visceral fat, is not merely a storage depot but a metabolically active organ that secretes pro-inflammatory adipokines such as leptin, resistin, and TNF-α, while downregulating protective factors like adiponectin (Lin and Li, 2021; Busebee et al., 2023). These shifts promote a systemic inflammatory milieu characterized by IL-6 and C-reactive protein, which are both independent predictors of diastolic dysfunction (Hildebrandt et al., 2023). Simultaneously, obesity impairs endothelial nitric oxide (NO) bioavailability through increased oxidative stress, mediated by NADPH oxidase-derived ROS (Busebee et al., 2023; Soták et al., 2025). This endothelial dysfunction leads to microvascular rarefaction, limiting myocardial perfusion and creating a hypoxic environment that activates fibrotic signaling cascades, such as TNF-α and TGF-β1 (Hildebrandt et al., 2023) as demonstrated in Figure 4.

In obese individuals, salt retention is exacerbated by RAAS overactivation, enhanced aldosterone secretion, and resistance to natriuretic peptides (Engeli et al., 2006; Kawarazaki and Fujita, 2016). Excess sodium is stored in interstitial compartments - particularly in skin, muscle, and potentially myocardium - via glycosaminoglycan-bound reservoirs, in a process that bypasses osmotic regulation (Sulyok et al., 2022; Sembajwe et al., 2023). This non-osmotic sodium storage triggers macrophage activation and promotes further inflammatory and fibrotic remodeling (Sulyok et al., 2022). Notably, mineralocorticoid receptor (MR) activation in the setting of high sodium and leptin levels has been shown to upregulate profibrotic gene expression in cardiac fibroblasts, leading to cardiac fibrosis, the hallmark substrate of HFpEF (Lavall et al., 2014; Barrera-Chimal et al., 2022; Geng et al., 2023).

Taken together, the obese-inflammatory HFpEF phenotype and salt sensitivity appear to be mutually reinforcing pathophysiological states, each amplifying the myocardial substrate for diastolic dysfunction. The identification of this interplay has significant clinical implications. Therapies targeting both metabolic inflammation and sodium handling - such as low-sodium diets, MRAs, SGLT2 inhibitors (Ma et al., 2024), and potentially anti-inflammatory agents - may offer particular benefit in this subgroup. Importantly, early identification through advanced imaging or biomarker profiling in high-risk individuals could facilitate preemptive interventions, preventing progression to overt HFpEF.

##### 1.1.2.2 Hypertensive-aging HFpEF phenotype

The hypertensive-aging HFpEF phenotype represents a clinically and mechanistically distinct subgroup, predominantly affecting older adults with increased arterial stiffness and concentric left ventricular (LV) remodeling (Samson et al., 2016; Zhang et al., 2024a). Aging is a well-established driver of both HFpEF and salt sensitivity, owing to cumulative alterations in renal, vascular, and neurohormonal regulation (AlGhatrif et al., 2017). Age-related nephron loss, diminished baroreflex sensitivity, and reduced responsiveness to natriuretic peptides impair the kidney’s ability to excrete sodium, thereby enhancing salt-induced volume expansion and pressure lability (AlGhatrif et al., 2017) (Figure 4). These ionic and hemodynamic perturbations promote subclinical myocardial remodeling, typified by increased LV stiffness and impaired relaxation (Samson et al., 2016). Indeed, studies in elderly salt-sensitive individuals have documented early evidence of LV remodeling - particularly concentric hypertrophy and increased relative wall thickness - even in the setting of preserved ejection fraction, structural changes that are characteristic of the early HFpEF phenotype (Li et al., 2022).

Complementing these cardiac changes are vascular alterations intrinsic to aging. A progressive increase in arterial stiffness, driven by degradation of elastin, collagen cross-linking, and accumulation of advanced glycation end-products (AGEs) (Zieman et al., 2005; Samson et al., 2016; AlGhatrif et al., 2017), leads to widened pulse pressure and augmented systolic load. These changes amplify early wave reflections, increasing myocardial workload and compromising coronary perfusion during diastole (Buffolo et al., 2022; Kim and Jo, 2024). Importantly, arterial stiffness is not merely a correlate but an independent predictor of HFpEF, and is consistently high in HFpEF patients (Mizuno et al., 2023; Kim and Jo, 2024). Furthermore, chronic activation of neurohormonal pathways - including angiotensin II, aldosterone, and endothelin - along with systemic inflammation and oxidative stress, fosters myocardial fibrosis through ECM remodeling and endothelial dysfunction (Lopez-Lopez et al., 2021; Li et al., 2022; Bailey and Dhaun, 2024). This pathophysiologic convergence of aging, salt sensitivity, and arterial stiffening provides a mechanistic explanation for the high prevalence and unique characteristics of HFpEF in the elderly population.

##### 1.1.2.3 Renal dysfunction-driven HFpEF phenotype

The renal dysfunction-driven HFpEF phenotype is increasingly recognized as a distinct clinical and pathophysiological subgroup, characterized by impaired natriuresis, fluid retention, and uremia-induced vascular injury (Bailey and Dhaun, 2024). In individuals with chronic kidney disease (CKD), reduced glomerular filtration and diminished sodium excretory capacity promote chronic volume overload, a key driver of elevated LV filling pressures and diastolic dysfunction (Kirkman et al., 2021; Ito et al., 2023). Notably, salt sensitivity shares this fundamental natriuretic defect, and the two conditions frequently coexist, especially in older adults and those with comorbid hypertension or diabetes (AlGhatrif et al., 2017; Bailey and Dhaun, 2024).

Recent advances in imaging, particularly sodium-23 magnetic resonance imaging (^23^Na-MRI), have provided direct evidence of interstitial sodium accumulation in CKD patients, even in the absence of overt volume overload (Schneider et al., 2017; Qirjazi et al., 2020; Akbari and McIntyre, 2023). This sodium retention is not simply intravascular but occurs within glycosaminoglycan-bound tissue compartments in the skin, muscle, and potentially myocardium (Schneider et al., 2017; Qirjazi et al., 2020; Ito et al., 2023). In both CKD and salt-sensitive individuals, such non-osmotic sodium storage acts as a stimulus for macrophage activation, oxidative stress, and the release of pro-fibrotic mediators (Ito et al., 2023). The cumulative effect is diffuse myocardial fibrosis, impaired LV compliance, and the progression toward the HFpEF phenotype.

Moreover, CKD is associated with uremia-related endothelial dysfunction, increased arterial stiffness, and impaired NO signaling - factors that further compromise coronary microvascular function and contribute to ventricular-vascular uncoupling (da Cunha et al., 2020). In parallel, salt-sensitive individuals exhibit enhanced MR signaling and blunted natriuretic peptide responsiveness, both of which exacerbate sodium retention and myocardial remodeling (Lavall et al., 2014; Geng et al., 2023). Together, these overlapping features reinforce the view that the renal HFpEF phenotype and salt sensitivity share common molecular and hemodynamic pathways, centered around impaired sodium homeostasis, systemic inflammation, and myocardial fibrosis.

This mechanistic link is supported by data from Schneider et al. (2017), who used ^23^Na-MRI in a cohort of patients with mild to moderate CKD. The study found a strong positive correlation between skin sodium content, systolic blood pressure, and LV mass, independent of total body water and blood pressure control. These findings suggest that tissue sodium accumulation may drive myocardial remodeling via mechanisms unrelated to classical hemodynamic load, reinforcing the concept that sodium dysregulation itself contributes to the structural underpinnings of HFpEF. In this context, CKD and salt sensitivity appear to represent converging biological states, both predisposing to myocardial fibrosis, impaired compliance, and the evolution of HFpEF.

##### 1.1.2.4 Female-predominant HFpEF phenotype

Lastly, the female-predominant HFpEF phenotype is increasingly recognized as a distinct clinical entity, particularly prevalent among postmenopausal women, who comprise a disproportionate share of HFpEF cases globally (Sotomi et al., 2021; Teramoto et al., 2022). This phenotype is tightly linked to the decline in estrogen levels following menopause, which alters vascular and renal physiology in ways that heighten salt sensitivity. Estrogen normally promotes natriuresis, enhances endothelial function, and attenuates sympathetic tone; its loss leads to increased vascular resistance, reduced NO bioavailability, and enhanced sodium retention (Schulman et al., 2006; Du et al., 2025) (Figure 4). As a result, postmenopausal women exhibit greater susceptibility to volume shifts and pressure overload, despite normotensive profiles in clinical settings (Schulman et al., 2006; Pedrotty and Jessup, 2015; Du et al., 2025). These structural and functional alterations occur without overt changes in systolic function, often eluding early detection by conventional diagnostic approaches. Additionally, the volume shifts also have potential to cause disproportionate rise in filling pressures, and might lead to symptomatic limitation even in the absence of overt congestion (Aziz et al., 2013).

Some imaging studies reveal disproportionately high myocardial remodeling and fibrosis burden in women (Elboudwarej et al., 2018), reflecting early structural changes that often precede overt functional decline and remain undetected by conventional diagnostic modalities. In some cases, women present with exertional dyspnea, preserved ejection fraction, and subtle hemodynamic abnormalities that belie the underlying myocardial remodeling (Elboudwarej et al., 2018). These findings align with other studies showing that salt-sensitive women - even without overt hypertension - are at greater risk of developing diastolic dysfunction and progressing to HFpEF (Masenga et al., 2025). This risk is further amplified by sex-specific differences in cardiac energetics, inflammatory signaling, and vascular stiffness, all of which worsen in the setting of sodium overload (Masenga et al., 2025).

Taken together, these overlapping phenotypes highlight the importance of considering salt sensitivity as more than a trait of blood pressure regulation. Rather, it appears to function as a trans-diagnostic modifier that exacerbates myocardial fibrosis and dysfunction across multiple HFpEF subtypes. Recognizing and stratifying this intersection could enhance both early detection and targeted management strategies, especially in demographic groups disproportionately affected by both conditions.

#### 1.1.3 Advanced imaging: bridging salt-induced myocardial pathophysiology with early clinical detection

The pathophysiological mechanisms underlying salt sensitivity culminate in a progressive, insidious accumulation of diffuse interstitial fibrosis. As noted earlier, these myocardial changes often occur in the absence of overt hypertension or structural heart disease, where cardiac remodeling is driven by complex inflammatory pathways and neurohormonal alterations rather than pressure overload alone (Morris et al., 1999; Ferreira et al., 2010; Sopel et al., 2011).

Despite emerging evidence linking salt sensitivity to myocardial fibrosis, the clinical identification of these early fibrotic changes remains a substantial challenge. Conventional diagnostic modalities such as echocardiography and electrocardiography, while useful in evaluating global cardiac function, lack the sensitivity to detect subtle interstitial changes in the ECM (Ciulla et al., 1997), particularly in the preclinical stages of myocardial remodeling. As a result, many at-risk individuals progress undetected, only to present later with irreversible myocardial damage or symptomatic heart failure.

This diagnostic gap underscores the urgent need for imaging modalities that align more closely with the biological underpinnings of salt-induced myocardial injury. Advanced imaging techniques - specifically T1 mapping on cardiac MRI and the emerging use of sodium ^23^Na-MRI - offer the potential to non-invasively detect diffuse myocardial fibrosis at a stage when intervention may still be both feasible and effective (Bottomley, 2016; Martin et al., 2022).

These technologies represent a critical evolution in cardiovascular imaging, enabling the visualization and quantification of myocardial changes that were previously confined to histological evaluation. In doing so, they serve not merely as diagnostic tools but as translational bridges - linking subclinical myocardial pathology to actionable clinical insight in salt-sensitive populations.

##### 1.1.3.1 ^23^Na-MRI: a glimpse into ionic pathobiology and its role in early myocardial fibrosis detection

^23^Na-MRI offers a unique capability to noninvasively visualize and quantify sodium distribution within biological tissues (Kopp et al., 2013; Christa et al., 2024). Unlike conventional proton (^1^H) MRI, which provides anatomical and functional data based on water content, ^23^Na-MRI leverages the magnetic properties of the sodium-23 nucleus (Martin et al., 2022; Christa et al., 2024). Due to its lower gyromagnetic ratio and abundance, ^23^Na MRI requires high-field strength systems (≥3T) and specialized radiofrequency coils to achieve adequate signal-to-noise ratios (Martin et al., 2022; Christa et al., 2024). Additionally, tailored imaging sequences, such as 3D radial or twisted projection imaging, are necessary to capture signals from this rapidly relaxing nucleus (Nielles-Vallespin et al., 2007). Though the spatial resolution of ^23^Na-MRI is lower than that of standard MRI, it is sufficient for mapping sodium concentrations in key compartments including the myocardium, skeletal muscle, and skin (Kopp et al., 2013). This modality provides insight into ionic homeostasis and allows for the distinction between osmotic and non-osmotic sodium storage - an especially relevant feature in salt-sensitive cardiovascular pathology. In clinical studies, hypertensive patients show significantly higher sodium content than normotensive controls, independent of water content - suggesting the presence of water-free sodium storage. These findings, as demonstrated by Kopp et al. (2013), support the role of tissue sodium deposition as an early marker of salt-sensitive hypertension and highlight the translational potential of sodium imaging for cardiovascular risk stratification.

However, although sodium imaging offers valuable molecular insight into sodium retention and myocardial fibrosis, its standalone clinical utility remains limited by technical and biological factors, for example, its inability to delineate infarcted regions (Ouwerkerk et al., 2008). Nonetheless, its relevance is enhanced when combined with high-resolution techniques such as T1 mapping (Ambale-Venkatesh and Lima, 2015) and complementary biomarker panels, which together enable a more comprehensive assessment of myocardial remodeling. When integrated with T1 mapping, ^23^Na-MRI facilitates a more complete characterization of both ionic and structural changes, which may be particularly relevant in salt-sensitive HFpEF phenotypes.

Though still in the investigational phase, ^23^Na-MRI may still have profound implications:

• Risk stratification: Identifying individuals with silent sodium overload and early fibrotic remodeling, well before symptoms emerge.
• Therapeutic monitoring: Evaluating the response to low-sodium dietary interventions, MRAs, ARBs, or SGLT2 inhibitors.
• Comparative effectiveness: Complementary use with T1 mapping may offer a comprehensive assessment of myocardial ionic, structural, and extracellular matrix changes.

Therefore, ^23^Na-MRI offers an unprecedented window into early-stage, pre-symptomatic myocardial pathology, particularly in populations with high salt sensitivity, laying the groundwork for individualized cardiovascular risk profiling and preventive therapy.

#### 1.1.4 Therapeutic strategies for salt-induced myocardial fibrosis: An evolving frontier

Despite growing recognition of salt sensitivity as a key contributor to myocardial fibrosis and HFpEF pathogenesis, therapeutic strategies specifically targeting salt-induced myocardial remodeling remain poorly defined. While several pharmacologic and lifestyle interventions are biologically plausible and have demonstrated benefits in broader HFpEF populations, their efficacy in reversing or halting salt-driven myocardial fibrosis has not been rigorously established.

##### 1.1.4.1 Mineralocorticoid receptor antagonists

MRAs include both steroidal agents, such as spironolactone and eplerenone, and newer nonsteroidal agents such as finerenone (Gregg and Navaneethan, 2022). Steroidal MRAs have shown modest clinical benefit in HFpEF in select subgroups, particularly those with elevated natriuretic peptides and evidence of fibrosis or diastolic dysfunction (Palanisamy et al., 2022; Sethi et al., 2024). Mechanistically, they mitigate aldosterone-driven sodium retention, inflammation, and myocardial fibrosis, supporting their relevance in salt-sensitive individuals (Kagiyama et al., 2007; Barrera-Chimal et al., 2022; Geng et al., 2023). Preclinical models demonstrate that MR blockade attenuates salt-induced cardiac hypertrophy and collagen deposition (Rickard et al., 2012). However, human trials such as TOPCAT reported mixed results, with regional variability in response and no definitive impact on reversing cardiac fibrosis (Pitt et al., 2014).

In contrast, finerenone, a nonsteroidal MRA with greater receptor selectivity and anti-inflammatory properties, has shown encouraging results in HFpEF and heart failure with mid-range ejection fraction (HFmrEF) populations (Akshay et al., 2025). In the FINEARTS-HF trial (2025), those who were enrolled within 1 week of a worsening heart failure event experienced a 26% relative reduction in the risk of cardiovascular death or recurrent decompensation with finerenone compared to placebo. A 21% risk reduction was also observed in patients enrolled within 3 months of a prior event. Importantly, these benefits were achieved without excess adverse events, suggesting that finerenone may be particularly effective in higher-risk patients during this early vulnerable period. However, as the trial did not stratify participants by salt sensitivity, the extent to which sodium-related mechanisms contributed to these benefits remains uncertain and warrants further investigation.

##### 1.1.4.2 Angiotensin receptor blockers

ARBs have been recognized for their efficacy in reducing afterload and preventing adverse cardiac remodeling in heart failure with reduced ejection fraction (Heidenreich et al., 2022). Recent evidence, however, suggests that their utility extends to patients with HFpEF, particularly those exhibiting salt-sensitive pathophysiology. Mechanistically, ARBs attenuate angiotensin II–mediated vasoconstriction, sodium retention, and fibrotic signaling cascades, including suppression of TGF-β (Tokuda et al., 2004; Burke et al., 2019). In animal models of pressure overload, subdepressor doses of candesartan reduced perivascular and interstitial fibrosis independent of blood pressure effects, primarily by downregulating monocyte chemoattractant protein-1 (MCP-1) and TGF-β expression (Tokuda et al., 2004). Complementing these findings, Imanishi et al. (2012) demonstrated in diabetic patients with early nephropathy that valsartan reduced salt sensitivity of blood pressure by restoring renal NO synthesis. The result of this was enhanced endothelial function, and lower oxidative stress - mechanisms closely linked to myocardial remodeling (Imanishi et al., 2012). In a clinical context, another study reported that ARB use in patients with obstructive hypertrophic cardiomyopathy was independently associated with reduced late gadolinium enhancement (LGE%) on cardiac MRI, indicating decreased myocardial fibrosis (Nie et al., 2024). Moreover, in a preclinical study by Burke et al. (2019), the combination of sacubitril/valsartan directly inhibited cardiac fibroblast activation and collagen deposition via restoration of protein kinase G signaling and inhibition of Rho-mediated myofibroblast transition. These converging lines of evidence support the potential of ARBs not only to reduce blood pressure but also to mitigate salt-driven myocardial fibrosis through pleiotropic effects on fibroinflammatory signaling and tissue remodeling. Despite these promising findings, clinical trials have yet to stratify HFpEF patients by salt sensitivity, leaving an important gap in translating these mechanistic benefits into precision-guided therapy.

##### 1.1.4.3 SGLT2 inhibitors

SGLT2 inhibitors have emerged as a cornerstone therapy in HFpEF, demonstrating consistent improvements in heart failure outcomes across trials such as EMPEROR-Preserved and DELIVER (Anker et al., 2021; Solomon et al., 2022). Their mechanisms extend beyond glycemic control, involving natriuresis, osmotic diuresis, reduced inflammation, and improved mitochondrial function (Wan et al., 2020; Yau et al., 2022). These properties suggest a theoretical benefit in salt-sensitive patients, where sodium and fluid handling are central to disease progression (Ma et al., 2024). However, there is no direct evidence to date that SGLT2 inhibitors reverse myocardial fibrosis, and their effects on sodium storage in non-osmotic tissue compartments (e.g., skin or myocardium) remain unexplored. Furthermore, most major trials did not assess or stratify participants by salt sensitivity, fibrosis burden, or non-osmotic sodium retention, limiting insights into disease-specific reversibility (Anker et al., 2021; Solomon et al., 2022).

##### 1.1.4.4 Dietary sodium restriction

Dietary sodium restriction is an intuitive and mechanistically sound intervention in salt-sensitive hypertension and HFpEF, especially considering the well-established role of sodium overload in promoting myocardial fibrosis. Small trials have demonstrated that lower sodium intake can reduce arterial stiffness, filling pressures, and oxidative stress in hypertensive HFpEF patients (Hummel et al., 2013). However, whether sodium restriction can reverse established myocardial fibrosis remains uncertain. In salt-sensitive individuals, tissue sodium is often non-osmotically stored in glycosaminoglycan-bound interstitial compartments, and may not be readily mobilized through acute dietary changes alone (Sulyok et al., 2022; Ito et al., 2023). The recently published SODIUM-HF trial (Ezekowitz et al., 2022) adds further nuance to this discussion. In this international randomized trial of ambulatory patients with chronic heart failure, a structured dietary sodium reduction to <1,500 mg/day did not significantly reduce the composite outcome of cardiovascular hospitalizations, emergency visits, or mortality over 12 months, compared with usual care. Despite successful sodium reduction in the intervention group, clinical events were not meaningfully different (HR 0.89; 95% CI 0.63–1.26; *p* = 0.53), suggesting that sodium restriction may not confer universal benefit in a heterogeneous heart failure population. These findings highlight the need for a phenotype-specific approach, as patients with demonstrable salt sensitivity and evidence of sodium-driven fibrotic remodeling may derive greater benefit. Long-term adherence, variability in salt responsiveness, and the complexity of tissue sodium dynamics continue to pose challenges to broad implementation. As such, sodium restriction may remain a supportive rather than standalone strategy in the management of myocardial fibrosis in HFpEF.

##### 1.1.4.5 The unmet need: targeted antifibrotic therapies

While the above therapies offer pathway-specific benefits, none are explicitly designed to reverse salt-induced myocardial fibrosis. Novel antifibrotic agents, such as galectin-3 inhibitors (Bouffette et al., 2023), TGF-β modulators, and anti-inflammatory compounds targeting macrophage activation (Biernacka et al., 2011; Navaneethabalakrishnan et al., 2024), are under investigation in other fibrotic conditions and may hold promise for cardiovascular application. Targeting the p38MAPK pathway has also shown promise in attenuating myocardial fibrosis in experimental models, with selective inhibitors such as SB203580 and PD-098059 demonstrating reductions in collagen deposition and improved cardiac function independent of blood pressure control (Ying and Sanders, 2002; Liu et al., 2005; Paw et al., 2021). Similarly, non-pharmacologic strategies such as structured low-sodium dietary interventions, combined with biomarker-guided therapy, could represent future precision-based approaches.

## 2 Conclusion

Salt-sensitivity represents a unique cardiovascular phenotype where excessive sodium intake leads to blood pressure elevation and myocardial fibrosis through both hemodynamic and non-hemodynamic pathways. The interplay between PRR and AT1R, and downstream mediators such as p38MAPK and TGF-β, provides a mechanistic framework for understanding how ionic imbalance, immune activation, and fibrotic signaling converge to drive adverse cardiac remodeling. Importantly, this fibrotic cascade may be activated even in the absence of overt hypertension, challenging traditional diagnostic paradigms. Innovative imaging tools such as ^23^Na-MRI with T1 mapping offer noninvasive windows into myocardial sodium handling and tissue composition, revealing early fibrotic changes that escape detection by standard modalities. Therapeutically, MRAs, ARBs, SGLT 2 inhibitors, and phenotypic-specific sodium modulation collectively offer a multifaceted strategy to attenuate myocardial fibrosis in salt-sensitive individuals and should be prioritized in phenotype-directed management.

### 2.1 What is known?

• Salt sensitivity is a significant risk factor for cardiovascular disease and hypertension-related morbidity.
• High dietary sodium contributes to blood pressure elevation, particularly in salt-sensitive individuals.
• Myocardial fibrosis is a key pathological substrate in cardiac remodeling and diastolic dysfunction.
• Traditional assessment of salt sensitivity relies on blood pressure response, with limited insight into tissue-level changes.

### 2.2 What is new?

• Salt-sensitive myocardial fibrosis may occur independently of blood pressure changes, via molecular and redox-sensitive mechanisms.
• High sodium intake activates MAPK pathways and promotes fibrotic gene expression, even in the absence of hypertension.
• Tissue sodium overload may contribute to early myocardial stiffening and fibrosis, potentially leading to HFpEF.
• ^23^Na-MRI enables noninvasive visualization of myocardial sodium accumulation, distinguishing osmotic from non-osmotic sodium storage.
• Emerging therapies targeting sodium compartmentalization and downstream fibrotic signaling offer novel treatment avenues beyond BP control.
